# Correction: ‘Inference of the drivers of collective movement in two cell types: *Dictyostelium* and melanoma’ (2016), by Ferguson *et al.*

**DOI:** 10.1098/rsif.2026.0986

**Published:** 2026-10-21

**Authors:** Elaine A. Ferguson, Jason Matthiopoulos, Robert Insall, Dirk Husmeier

**Affiliations:** ^1^ School of Biodiversity, One Health & Veterinary Medicine, College of Medical, Veterinary & Life Sciences, University of Glasgow, Glasgow, UK; ^2^ School of Mathematics and Statistics, University of Glasgow, Glasgow, UK; ^3^ Division of Cell & Developmental Biology, University College London, London, UK; ^4^ Cancer Research UK Scotland Institute, Bearsden, Glasgow, UK

*J. R. Soc. Interface* **13**, 20160695. (Published online 1 October 2016). (https://doi.org/10.1098/rsif.2016.0695)

The following text corrects the manuscript by Ferguson *et al.* [1].

In the caption of figure 2a, it should state that this image is a reproduction from [2] (reference 3 in the original article):

‘(a) Image taken 40 h into the melanoma cell movement assay (see (e) for corresponding cell density estimate). Reproduced from figure 1g of [3] (CC BY 4.0)’.

Figure 1a was generated as part of the study described in [3] (reference 7 in the original article), but was not previously published.
